# Steroid receptor coactivators: from basic research to translational opportunities

**DOI:** 10.1210/endrev/bnag003

**Published:** 2026-02-20

**Authors:** Yosef Gilad, Sang Jun Han, David M Lonard

**Affiliations:** Department of Molecular and Cellular Biology, Baylor College of Medicine, Houston, TX 77030, USA; Department of Molecular and Cellular Biology, Baylor College of Medicine, Houston, TX 77030, USA; Nuclear Receptor, Transcription and Chromatin Biology Program, Dan L. Duncan Cancer Center, Baylor College of Medicine, Houston, TX 77030, USA; Department of Molecular and Cellular Biology, Baylor College of Medicine, Houston, TX 77030, USA; Nuclear Receptor, Transcription and Chromatin Biology Program, Dan L. Duncan Cancer Center, Baylor College of Medicine, Houston, TX 77030, USA

**Keywords:** steroid receptor coactivator (SRC), nuclear receptor coregulator, therapy resistance, immunotherapy

## Abstract

The concept of nuclear receptor (NR) coregulation was proposed nearly 2 decades before it was experimentally validated. According to this model, NRs—executors of a vast array of transcriptional programs—do not act independently but are governed by a network of regulatory proteins that either activate or repress their biological function. Nuclear receptors identify the genes to be regulated. However, coregulators ultimately serve as the true controllers of transcriptional outcomes. They recruit cofactors and coordinate the activity of transcriptional complexes, thereby shaping NR-mediated gene expression beyond NRs’ intrinsic functionality. The steroid receptor coactivator (SRC) family is the most extensively studied and functionally dominant group of NR coregulators, modulating nearly all gene activities. The pleiotropic biological roles of SRCs—spanning key physiological and pathological processes—make them compelling therapeutic targets. Here, we outline the evolution of the coregulation concept, which reached a critical milestone with the discovery of the SRC family. We highlight the central roles of SRCs in both physiology and oncology and trace the development of therapeutic strategies aimed at targeting these proteins.

## Essential points

- The transcriptional activity of NRs relies on and is regulated by coregulator proteins
- The SRC family comprises 3 paralogous proteins—SRC-1, SRC-2, and SRC-3—that represent the most extensively studied coactivators and participate in key physiological processes including reproduction, metabolism, immunity, and oncogenesis
- SRCs were long considered “undruggable” due to their large size and lack of a well-defined ligand-binding pocket, until the identification of the first SRC-targeting compound, gossypol, in 2011
- The development of synthetic, drug-like small-molecule modulators—both stimulators and inhibitors—enabled exploration of SRCs as potential therapeutic targets in cancer
- The immunomodulatory role of SRCs is most consequentially demonstrated by the finding that inhibition of SRC-3 in Tregs reprograms the tumor immune environment, enhances local inflammation, promotes cytotoxic lymphocyte infiltration, and results in tumor eradication in mouse models of solid tumors

**Figure 1:**
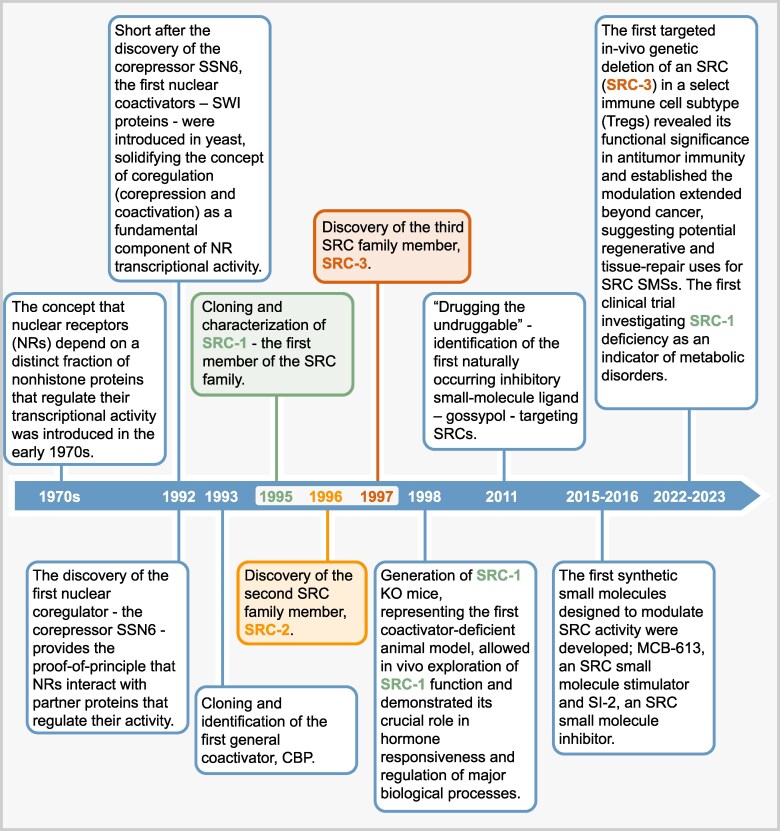
Timeline highlighting key milestones in the discovery and characterization of SRCs, from the emergence of the coregulation concept to the development of SRC-targeted therapeutics.

Nuclear receptors (NRs) are a family of transcription factors (TFs) that regulate a broad range of physiological processes (1). For many years, it was believed that all members of the NR family require ligand (hormone) binding to become biologically active. However, the identification of structurally related proteins that lack known ligands (2, 3), thus referred to as “orphan” receptors (3, 4), changed this paradigm. It became clear that NRs actually comprise a much larger group of TFs sharing a high degree of homology and that not all members necessarily depend on hormone molecules—or indeed any ligand at all—for their activity (5). In fact, of the 48 NRs identified in humans, several—such as NR4A1 (Nur77), PNR (NR2E3), and COUP-TFII (NR2F2), which play essential physiological roles in processes such as development and immunity (6-8)—remain classified as “true orphans,” distinguishing them from “adopted orphans” for which endogenous ligands have been identified (1, 5, 9). Importantly, mutations in certain ligand-dependent NRs, particularly estrogen receptor α (ERα), can confer ligand-independent activity, including the aberrant recruitment of cofactor proteins that supports unregulated transcription and is associated with cancer progression (10-12). However, under normal physiological conditions—whether ligand-dependent or orphan—NRs cannot drive transcription independently. The idea that for a full scale of their activity, NRs rely on a distinct family of proteins was introduced in the early 1970s (Fig. 1) (13-15) but was not accepted without initial skepticism (16). Nearly 2 decades after this hypothesis was proposed, the first coregulators—a corepressor and coactivator—were cloned (17, 18). These seminal discoveries opened the door to a plethora of publications, and in fact, it became clear that the entire scope of NR activity is regulated by “partner” proteins that can either activate or repress NR-mediated transcription. It did not take long after these pioneer discoveries for coregulators to be recognized as critical components of gene regulation (19). This ultimately led to the identification of the first—and arguably most dominant—coactivator family: the steroid receptor coactivators (SRCs), initiated by the discovery of the first family member, SRC-1 (20). Since the discovery and identification of their biological activity, the 3 members of the SRC family—SRC1/2/3—have been established as central pillars of gene regulation that coordinate the assembly of multiprotein transcription complexes and facilitate the recruitment of key chromatin-modifying enzymes such as CREB-binding protein (CREBBP/CBP), p300 (EP300), and coactivator-associated arginine methyltransferase 1 (CARM1) (21). By mediating these molecular interactions, the SRCs exert widespread control over gene expression, with broad implications for both physiological function and disease states (22). The central role of SRCs in diverse physiological processes—mediated through their interactions with numerous NRs and other TFs—has positioned them as attractive platforms for therapeutic intervention with broad clinical potential. However, SRCs were long regarded as “undruggable” targets due to their large size, intrinsic structural disorder, and absence of a conventional ligand-binding pocket (16). Initial efforts to overcome the perceived “undruggability” of SRCs focused on disrupting their protein–protein interactions with NRs, rather than by developing compounds that act on SRCs as primary molecular targets (23-25). The longstanding challenge in developing pharmacological strategies that act directly on SRCs was eventually met through the use of high-throughput screening technology combined with luciferase reporter assays, which were designed to detect changes in SRC-mediated transcriptional activity and protein abundance (21, 26). This approach led to the discovery of the first-in-class synthetic SRC small-molecule inhibitor (SMI) (27), as well as the small-molecule stimulator (SMS) (28) for applications in cancer treatment. These breakthroughs paved the way for the development of second-generation SRC SMIs and SMSs and laid the foundation for additional applications beyond cancer treatment (29).

In this review, we first outline how NR coregulation has evolved into a central discipline within modern molecular-endocrine biology. We then focus on the SRC family—arguably the most dominant and functionally versatile family of coregulators—and describe their major physiological and oncogenic roles that underscore their significance as therapeutic targets. We further describe the discovery and development of small-molecule inhibitors and activators of SRCs, which were long considered undruggable oncogenes. Finally, we highlight emerging opportunities that arise from modulating SRC activity beyond tumor suppression, particularly in achieving therapeutically beneficial immune modulation.

## Steroid receptor coactivators: background

**Figure 2:**
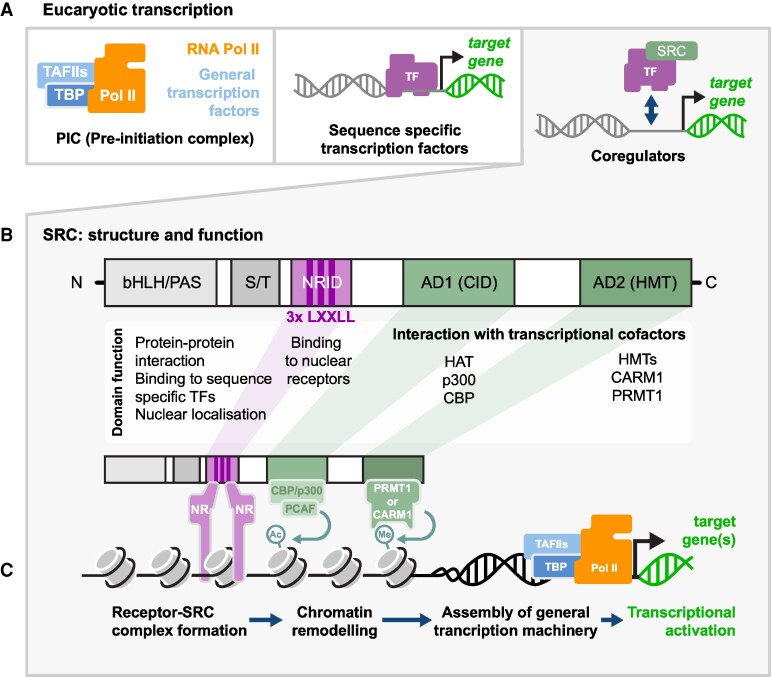
Functional domains of the SRCs: (A) Eukaryotic transcription as a multilayered, hierarchical process, with coregulator proteins at its highest regulatory level. (B) The molecular structure of SRC family members contains several conserved structural domains: a bHLH/period aryl hydrocarbon/simple-minded (bHLH-PAS) domain, a serine/threonine-rich (S/T) domain, a nuclear receptor interaction domain (NRID), a histone acetyltransferase (HAT) domain, and an HMT domain. (C) SRCs act as scaffold proteins to integrate the interaction of NRs and chromatin remodelers with the genome and promote assembly of the Pol II transcriptional machinery.

The most fundamental determinant of eukaryotic transcription is the interaction of RNA polymerase II (Pol II) with the genome. Pol II is a 12-subunit protein complex that functions in concert with general transcription factors (GTFs) to assemble into an even larger preinitiation complex, comprised of several dozen subunits (30, 31). This degree of complexity provides the transcription process with a high level of regulatory precision. Beyond the core preinitiation machinery, sequence-specific TFs further complicate the process. These factors interpret upstream signals and chromatin context to exert gene-specific control, ultimately dictating the fate of transcription at the level of individual genes (32). Furthermore, as was first proposed in the early 1970s, the regulatory landscape of gene expression involves an additional class of proteins, coregulators, that modulate the activity of TFs (Fig. 2). Originally, the hypothesis of the existence of *helper* proteins, that partner with NRs, was introduced in the context of the activity of the progesterone receptor (PR)—as essential facilitators of its hormone-dependent transcriptional activity (14, 15). These proteins were first termed “acceptor proteins” or “acceptor molecules” to reflect their role in determining the nature of functional hormone receptor binding to its tissue-specific chromatin “acceptor cites” (13, 33, 34). Ultimate proof for the existence of NR coregulators came 2 decades later with 2 publications that identified the first corepressor and the first coactivator of NRs. The proof of principle that NRs are interacting with partner proteins that regulate their activity was first exemplified by the repressor protein SSN6; by binding to the TAF1 domain of the ER, SSN6 suppresses the hormone-dependent transcriptional activity of ER and PR. Mutations in SSN6, that disrupted its protein–protein interaction capability, led to increased hormone-dependent activity of both ER and PR (17). These findings suggested a dual role for the hormone: it not only promotes DNA binding but also induces a conformational change of the NR (ie, ER/PR) that antagonizes the activity of a corepressor protein (eg, SSN6). Shortly after this foundational demonstration of NR coregulation, another study reported that transcriptional activity of rat glucocorticoid receptor (GR) in yeast is enhanced upon its interaction with SWI proteins. Coprecipitation of the GR complex with SWI proteins was detected in wild-type (WT) yeast extracts but was absent in extracts from SWI–mutant strains, which also exhibited impaired GR-mediated transcriptional activation (18). These 2 seminal studies established the concept that NRs rely on “helper proteins” for the regulation of their transcriptional activity. This group of proteins, known as coregulators, includes coactivators and corepressors, which regulate NRs by subjecting them to either positive or negative regulation of their transcriptional activity (19). More than 30 years after the discovery of the first coregulators, several hundred have now been reported in the literature, with more likely to be discovered in the future (35, 36). Within the NR coregulator family, SRCs comprise one of the most important groups, acting as integrators of transcriptional activity not only of NRs but also of other TFs as well. SRC-1 was among the first coregulators to be cloned (20) after the discovery of the general coactivator/integrator CBP (37). Initially identified as an ER-associated protein, it was found to bind ER in a specific and ligand-dependent manner, implicating it as a central mediator of ER-dependent transcriptional activity (38). Subsequent cloning and characterization revealed SRC-1 to be a common coactivator that interacts with multiple NRs, including PR, GR, ER, TR, and Retinoid X Receptor (RXR) (20). Later studies further expanded its functional scope by demonstrating its ability to coactivate non-NR transcription factors, such as nuclear factor-kappa B (NF-κB), thereby positioning SRC-1 as a broadly acting coactivator (39-41). The discovery and characterization of SRC-1 was followed shortly by the cloning and identification of TIF2/SRC-2 (42) and RAC3/ACTR/SRC-3 (43-46), establishing the SRCs as the first characterized coactivator family and revealing significant structural similarity among the 3 members (45, 47). Of note, while SRCs are part of the broader nuclear coactivator (NCOA) family—comprising 7 human coregulators (NCOA1-7) (48)—they represent a distinct subgroup of 3 closely related members. All SRCs have a molecular weight of ∼160 kDa and share several conserved structural domains (Fig. 2B and 2C); the most conserved domain is the basic helix-loop-helix (bHLH)/Per/Arnt/Sim (bHLH-PAS) domain, which is located at the N-terminus and shares ∼60% amino acid identity between the SRC family members (46). bHLH-PAS is required for the protein–protein interaction capability of the SRCs (49-51)—including interactions with specific TFs (52, 53)—and it also contains a bipartite nuclear localization signal that directs the SRCs into the nucleus. The central region of the SRCs contains 3 LXXLL α-helical motifs (X represents any amino acid) with flanking sequences around them that together form the NR interaction domain, which is required for the activating or repressor interactions of the SRCs with their target NRs (54). The α-helical amphiphilic LXXLL motifs are essential for the SRCs to interact with NRs (55), while the flanking sequences dictate the strength and specificity of binding (56-58). The 2 activation domains, AD1 and AD2, are located at the C-terminus of SRCs and serve as key mediators of SRC interactions with essential transcriptional cofactors; the AD1 domain primarily recruits histone acetyltransferases such as p300 and CBP, while AD2 engages histone methyltransferases (HMTs) such as CARM1 and PRMT1 (21, 58, 59). Accordingly, AD1 is commonly referred to as the CBP-interacting domain, and AD2 is known as an HMT domain.

Due to their modular multidomain architecture, SRCs are ideal bridging molecules capable of simultaneously interacting with NRs, other TFs, and essential coregulatory elements such as GTFs and histone–chromatin remodelers (60). This enables SRCs to function as an epicenter of Pol II transcriptional complexes and to facilitate histone modifications critical for the regulation of gene expression. Moreover, it has been shown that the SRC-1 and SRC-3 have an intrinsic acetyltransferase activity (45, 61). This finding highlights an additional layer of functional flexibility in SRCs. Further versatility arises from posttranslational modifications (PTMs), which enhance the interaction specificity of SRCs and broaden the amplitude of their coregulatory activity. Among these, phosphorylation is a key PTM observed across all SRC family members. Generally, phosphorylation enhances SRC interactions with NRs, resulting in increased transcriptional activity, which in some cases has been associated with a potential risk of tumorigenesis (62, 63). Other PTMs such as ubiquitination, SUMOylation, and acetylation have also been observed. These modifications regulate SRC stability, cellular concentration, and overall activity, adding further complexity to their regulatory network (64). Altogether, the ubiquitous expression of SRCs, their ability to recruit a broad spectrum of regulatory proteins, and the added diversity of their activity conferred by PTMs position them as central players in gene expression regulation, ultimately reflecting their broad significance in human physiology and disease.

## SRCs in physiology

Steroid receptor coactivators are key regulators of major physiological and pathological processes, including reproduction, metabolism, immunity, and cancer. Although these processes are often interconnected and cannot be strictly compartmentalized, for the structural clarity of this review, we will begin by outlining the most well-established roles of SRCs in reproduction, metabolism, and immunity, followed by a stand-alone section describing their roles in hormone-related malignancies.

### Reproductive system

**Figure 3:**
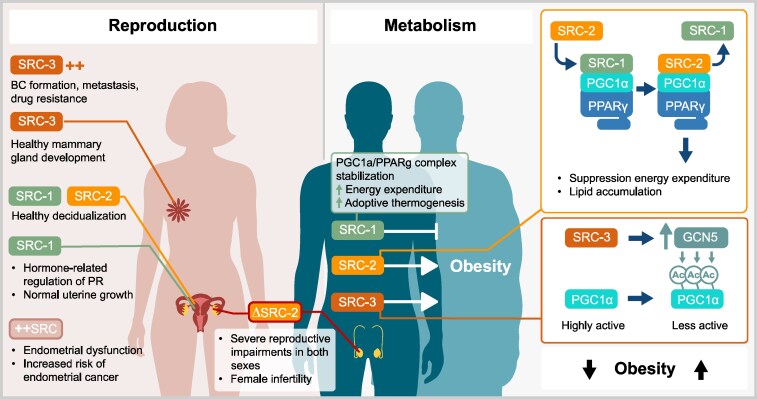
SRC functions in the reproductive system and lipid metabolism. Reproductive system: SRC-1 and SRC-2 are critical for hormone-dependent uterine growth and decidualization. Loss of SRC function impairs fertility, while their aberrant overexpression contributes to endometrial hyperplasia and increases cancer risk. SRC-3 supports normal mammary gland development, and its overexpression is strongly linked to ER-positive BCs. Lipid metabolism: SRC-1 promotes energy expenditure and adaptive thermogenesis by stabilizing the PGC-1α/PPARγ complex, thereby protecting against obesity. In contrast, SRC-2 and SRC-3 suppress energy expenditure and promote lipid storage. SRC-2 competes with SRC-1 for binding to the PGC-1α/PPARγ complex, antagonizing SRC-1's anti-obesity effects. SRC-3 enhances PGC-1α acetylation through upregulation of GCN5, leading to reduced PGC-1α activity.

All 3 SRCs play a significant role in the reproductive system’s development and function (Fig. 3). SRC-1 is an essential mediator of ER and PR activities in the uterus. Studies in genetically engineered mice revealed the primary role of SRC-1 in steroid hormone-related gene expression in the uterus, involving both PR and ER (65, 66). These NRs both are key regulators of decidualization—a critical process for normal embryogenesis, characterized by the rapid proliferation of endometrial stromal fibroblasts followed by their differentiation into decidual cells that form the specialized inner uterine lining that supports and immunologically protects the developing embryo (67). While SRC-1 knockout (KO) does not cause complete infertility, it is characterized by a failure to develop a full decidual response (65), underscoring its indispensable role in the regulation of PR and ER during early pregnancy. In addition to full-length SRC-1, a truncated isoform of SRC-1 also plays a significant role in endometrial biology. The SRC-1 isoform, which is generated through a matrix metalloprotease 9 (MMP-9)-mediated cleavage, retains key transactivation domains (AD1 and AD2) and promotes the survival of ectopic endometrial cells by blocking tumor necrosis factor alpha (TNFα)-induced apoptosis (68). It is implicated in endometriosis progression through its interaction with TFs such as ERβ and SF-1 (68). Interestingly, while this isoform supports lesion persistence under normal conditions, its overexpression—such as that induced by bufalin—can disrupt the ERβ/SRC-1 isoform axis and trigger proteasomal degradation of ERβ, ultimately suppressing lesion growth associated with cellular stress (69). These findings highlight the dual, context-dependent role of the SRC-1 isoform in endometrial biology and underscore its potential as a therapeutic target in endometriosis (70).

Steroid receptor coactivator 2 deficiency results in even more severe reproductive impairments than SRC-1 deficiency, highlighting its critical role in reproductive function across both sexes. In males, SRC-2 deletion causes hypofertility due to impaired spermatogenesis and testicular degeneration (71). Global SRC-2 KO in female mice also leads to infertility (71), which was shown—through tissue-specific SRC-2 KO models—to result in an insufficient decidual response and impaired embryo implantation (47, 72). SRC-2 plays a key role in the regulation of the PR-driven transcriptional program, which is essential for the proper development of decidual cells (73). While loss of SRC-2 impairs fertility, aberrant overexpression of SRC-2 is also linked to pathological reproductive outcomes and increased cancer risk. Notably, endometrial biopsies from patients with polycystic ovary syndrome (PCOS)—a condition associated with infertility and elevated risk of endometrial cancer—have shown increased SRC-2 expression (74-76). In a transgenic mouse model with conditional expression of human SRC-2, elevated SRC-2 levels in the endometrium have been shown to cause endometrial dysfunction and impair the decidualization process (77). Collectively, these findings suggest that dysregulated SRC-2 expression contributes not only to endometrial pathologies that lead to infertility but also to an elevated risk of developing hormone-dependent cancers.

While SRC-1 functions as a key coactivator for ER and PR in the uterus, SRC-3 serves as the primary coactivator for these crucial NRs in mammary luminal epithelial cells (78, 79). Although SRC-3 deficiency in male mice did not severely impact reproduction function, female mice lacking SRC-3 exhibit a significant decrease in estrogen levels associated with several reproductive system-related abnormalities, including delayed puberty, impaired reproductive functions, and defective mammary gland development (79). Moreover, female SRC-3 KO mice show significantly reduced alveolar development in the mammary gland following combined estrogen and progesterone stimulation, indicating that SRC-3 is essential for progesterone-mediated cell proliferation and glandular differentiation during mammary alveologenesis (79). Similar to SRC-2, abnormal overexpression of SRC-3 may drive endometrial dysfunction and act as a shared pathological mechanism connecting infertility with hormone-dependent cancers (74-76, 80).

The reproductive system is among the most dependent on proper function of all 3 SRC family members, which is unsurprising given the central role of SRCs as integrators of hormone responses. While SRC deficiencies result in severe reproductive defects and even infertility, their overexpression in reproductive organs is closely associated with dysregulated function and an increased risk of malignancies. Therefore, balanced expression of SRCs is essential for maintaining normal reproductive physiology.

### Metabolism

Steroid receptor coactivators play diverse roles in regulating metabolic pathways across multiple tissues, including the brain, liver, and muscle (81-83). However, their function as metabolic regulators is most extensively studied in adipose tissue, where they are essential for maintaining energy homeostasis (Fig. 3). Although both are adipose tissues, brown adipose tissue (BAT) and white adipose tissue (WAT) fulfill distinct metabolic roles: BAT specializes in energy expenditure via nonshivering thermogenesis, whereas WAT primarily functions in energy storage and endocrine regulation (84). SRCs serve as key modulators of the balance between BAT and WAT, coordinating thermogenic and lipogenic programs, playing distinct but also overlapping roles in regulating lipid metabolism. SRC-1 promotes energy expenditure and adaptive thermogenesis, primarily through stabilization of the peroxisome proliferator-activated receptor gamma (PPARγ) complex with its coactivator proliferator-activated receptor gamma coactivator-1 alpha (PGC-1α) (PGC-1α/PPARγ complex) in BAT (85). SRC-1 KO mice exhibit reduced energy expenditure and are prone to obesity, particularly under a high-fat diet (HFD) (85). Within the hypothalamus, SRC-1 associates with phosphorylated STAT3 to enhance leptin-driven transcription of proopiomelanocortin (POMC). The melanocortin peptides derived from POMC then activate the melanocortin-4 receptor (MC4R), leading to reduced food intake, thereby suggesting a protective role of SRC-1 against obesity (86). Supporting these observations, it has been reported that humans carrying SRC-1 variants that lead to SRC-1 deficiency exhibit complex metabolic syndromes, including childhood hyperphagia, adipose tissue fibrosis, and severe obesity (87). Recently, patients with rare SRC-1 variants and severe obesity had participated in phase 2 clinical trials of setmelanotide (88), an MC4R agonist approved for chronic weight management in POMC and leptin receptor deficiencies (89, 90).

Steroid receptor coactivator 2 (TIF2), the favored binding partner of the PGC-1α/PPARγ complex, antagonizes SRC-1 function, ultimately suppressing energy expenditure, thereby facilitating lipid accumulation and, under HFD, promoting obesity (85). HFD lowers SRC-1 levels in WAT and BAT (85), while aging also decreases its expression in WAT of both mice and humans (91), potentially contributing to age-related metabolic dysfunction. Double KO (DKO) of SRC-1 and SRC-3 in mice results in impaired development of BAT, lipid storage defects, disrupted energy expenditure, and mitochondrial uncoupling. These phenotype outcomes in the DKO mice are associated with downregulation of critical PPARγ target genes essential for these processes, such as UCP1/2 and AOX1, underscoring the importance of cooperative SRC-1/3 coregulation in fundamental metabolic functions (92). Interestingly, unlike SRC-1 KO mice, that were prone to develop obesity when fed with HFD (85), DKO mice, despite increased energy intake and disrupted thermogenesis, were lean and resistant to developing obesity. These contradictory observations were reasoned by the hyperactivity and high metabolic rate of the DKO mice (92), which in itself should be a subject for further exploration. Indeed, although partially, this has been explained by a study showing that SRC-3 is a positive regulator of the PGC-1α acetyltransferase GCN5. Accordingly, SRC-3 deficiency leads to downregulation of GCN5 expression, resulting in a deacetylated, active form of PGC-1α—a key regulator of PPARγ. Consequently, deacetylated PGC-1α-driven upregulation of PPARγ activity in SRC-3-deficient mice leads to increased mitochondrial function and enhanced energy expenditure (93). Even though SRC-1 plays a significant role in energy expenditure—unlike SRC-2 and SRC-3, which promote energy storage and reduce thermogenesis—it did not show a significant impact on adipogenesis. In contrast, single knockdown (KD) of SRC-2, SRC-3, or both significantly affected adipogenesis, implying critical, and, to some degree, overlapping roles for SRC-2 and SRC-3 in adipose tissue development, in addition to their function in energy storage (94).

Together, the SRCs form a complex and counterbalanced regulatory network that governs metabolic programming within adipose tissue, where each SRC shapes tissue function in response to nutritional and physiological cues. SRC-1 primarily promotes energy expenditure, whereas SRC-2 and SRC-3 support energy storage and adipogenesis. While adipose tissue remains the most extensively studied and illustrative system for the metabolic roles of SRCs, these coactivators also play important metabolic roles in other tissues, such as muscle, liver, and brain, with specific roles in amino acid and carbohydrate metabolism. These diverse functions have been the focus of several dedicated and comprehensive reviews (22, 81, 95).

### Immunity

In immunology, as in other physiological processes, the pleiotropic nature of SRCs is evident by their ability to function either synergistically or independently; they can compensate for one another's roles or display competing biological activities (96). The role of SRCs in immunology includes regulation of inflammatory processes, immune cell proliferation, fate determination, and function.

#### In Th17 and Tregs

**Figure 4:**
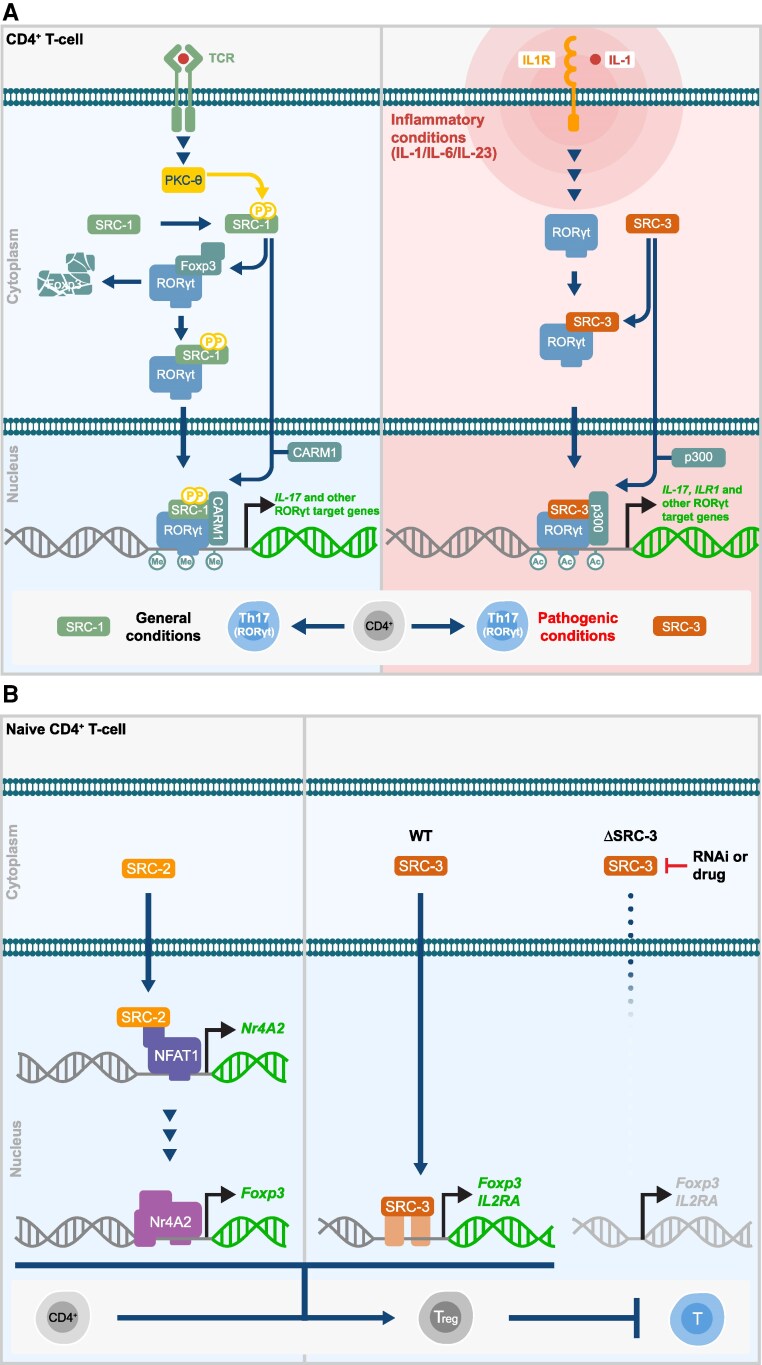

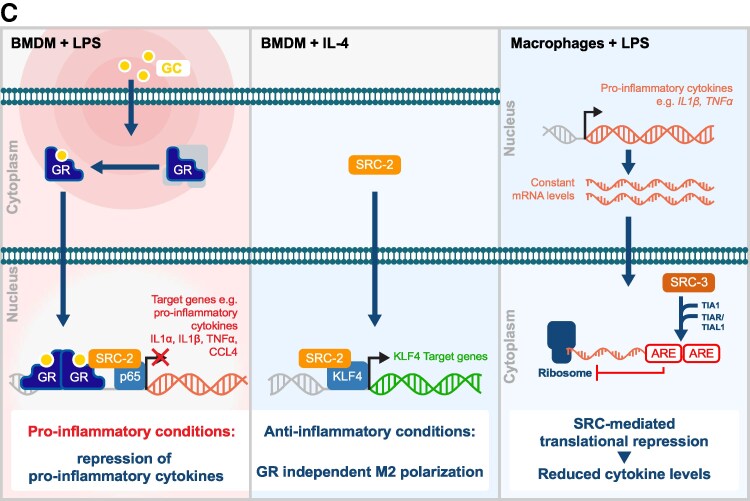
SRCs in the regulation of CD4^+^ T-cell fate and function and macrophage polarization: (A) SRC-1-RORγt interaction drives the predominance of Th17 cells over Tregs: When phosphorylated by PKC-θ, SRC-1 replaces FOXP3 in the RORγt complex, resulting in FOXP3 degradation and promoting the Th17 cell phenotype (left). SRC-3 promotes pathogenic inflammation by coactivating RORγt-driven expression of Th17 cell genes through a signaling pathway mediated by the IL-1–IL1R axis (right). (B) SRC-2 promotes the differentiation of Treg cells from CD4^+^ T cells: SRC-2 coactivates NFAT1, which drives the expression of Nr4a2, a key regulator of FOXP3 expression (left). SRC-3 is highly expressed and plays an important role in Treg cell biology. SRC-3 inhibition results in lower transcript levels of hallmark Treg genes, including FOXP3 and IL2RA, impairing a signature Treg function—the suppression of conventional T-cell proliferation (right). (C) Direct effects: under pro-inflammatory conditions, SRC-2 interacts with GR to suppress the NF-κB-driven inflammatory transcriptional program (left). Under anti-inflammatory conditions, SRC-2 coactivates KLF-4 to promote transcription of anti-inflammatory genes, establishing a homeostatic environment (middle). Under pro-inflammatory conditions, SRC-3 reduces the protein levels—but not transcript levels—of inflammatory cytokines in macrophages (right).

Th17 cells play a vital role in immune defense against extracellular pathogens and cancer, but they are also major contributors to chronic inflammation and autoimmune diseases (97). The subdifferentiation of naive CD4^+^ T cells into the different T helper (or Treg) subpopulations is a highly dynamic process that is heavily dependent on the biological context and governed by a complex signal network transduced primarily, but not exclusively, by cytokines (98, 99). SRC-1 and SRC-3 play an important role in determining fate commitment of CD4⁺ T cells toward the Th17 phenotype through interactions with RORγt (Fig. 4A). Specifically, SRC-1 promotes Th17 differentiation by directly activating RORγt and recruiting CARM1 to establish a permissive chromatin structure at the *IL17A* promoter, both dependent on PKC-θ-mediated phosphorylation of SRC-1 at serines 1271/1272. Mechanistically, phosphorylated SRC-1 displaces FOXP3 from the RORγt–FOXP3 complex, promoting FOXP3 degradation and shifting differentiation toward the Th17 lineage (100). SRC-3 also regulates Th17 gene expression through coactivation of RORγt. However, unlike SRC-1—whose function in Th17 differentiation is driven by protein kinase C theta (PKC-θ)-mediated T cell receptor (TCR) signaling—SRC-3 exerts its impact on Th17 fate determination via the inflammatory interleukin (IL)-1–IL1R1 signaling axis (Fig. 4A) (101). Under inflammatory conditions, SRC-3 recruits the histone acetyltransferase p300, promoting chromatin accessibility at key Th17 gene loci *IL17* and *IL1R1* (101). Accordingly, deletion of SRC-3 in T cells impairs the differentiation of Th17 cells under inflammatory but not under nonpathogenic conditions. This highlights the dual role of SRCs, both as transcriptional regulators of RORγt and as bridging proteins that integrate extracellular signals with epigenetic remodeling to drive the acquisition of Th17 cell phenotype. Although SRC-1 and SRC-3 both play pivotal roles in the regulation of Th17 cell differentiation, their functions diverge in the context of pathogenic vs nonpathogenic Th17 subsets. SRC-1 is broadly involved in Th17 differentiation and is regulated via TCR-PKC-θ-dependent signaling, promoting the expression of a general Th17 signature cytokine, IL17A. In contrast, SRC-3 exerts its function downstream of IL-1–ILR1 signaling and is specifically associated with the acquisition of a pathogenic effector phenotype of Th17 cells.

Tregs are physiologically antagonistic to Th17 cells. Their normal immunosuppressive function is critical for maintaining immune homeostasis, and loss of Treg suppressive activity can result in severe autoimmune disorders (102). The balance between Th17 and Tregs is dynamic and shaped by the surrounding cytokine milieu (103). Cancer cells frequently exploit Treg-mediated immunosuppression to evade immune responses, and high Treg infiltration often correlates with poor clinical outcomes (104). SRCs play diverse roles in both Th17 and Treg cells, as well as in regulating the balance and fate determination between these 2 lineages. In contrast to SRC-1, which skews the Treg–Th17 balance toward Th17 differentiation by counteracting FOXP3-mediated suppression (100), it has been shown that SRC-2 plays a critical role in the differentiation of naive CD4⁺ T cells into Tregs *in vitro* under transforming growth factor beta stimulation (105). Furthermore, unlike control FOXP3⁻ CD4⁺ T cells, those with FOXP3-dependent SRC-2 conditional KO (cKO) failed to induce FOXP3 expression *in vivo*. As a result, mice with SRC-2-deficient Tregs exhibit markedly elevated levels of pro-inflammatory cytokines compared with control mice and bear a higher risk of developing autoimmune disorders. Consistently, aged mice with SRC-2 KO Tregs are prone to spontaneous development of autoimmunity, accumulation of pro-inflammatory T cells in the lungs, and associated tissue damage. On a mechanistic level, cKO of SRC-2 in Tregs leads to downregulation of the orphan NR Nr4a2—a critical TF for Treg identity and function (106). Chromatin Immunoprecipitation-quantitative PCR (ChIP-qPCR) and immunoprecipitation studies revealed that SRC-2 is recruited by NFAT1 to the Nr4a2 promoter, suggesting that SRC-2 is required for proper Nr4a2 expression and, consequently, for maintaining Treg differentiation and function (Fig. 4B) (105). SRC-3 is enriched in Tregs, and its expression in these cells is significantly higher compared with other lymphocyte subsets, as shown by analysis of publicly available datasets and gene set enrichment analysis (107). Consistent with these bioinformatic observations, analysis of human samples revealed significantly elevated SRC-3 transcript levels in Tregs compared with the bulk CD4⁺ T-cell population, underscoring its biological relevance in this immune subset (107). Functional studies showed that both RNA interference-mediated KD and pharmacological inhibition of SRC-3 in human Tregs significantly reduced the expression of hallmark Treg genes, such as *FOXP3* and *IL2RA*, at transcript and protein levels (Fig. 4B).

#### In macrophages

Macrophages perform a wide range of physiological functions (108, 109) but their most critical roles are in innate immunity and the regulation of inflammatory responses (110, 111). Through their key position as major integrators of inflammatory responses, macrophages serve as central mediators of both pathological processes and protective and homeostatic functions (112). The 3 SRC family members modulate macrophage functions in both pathological and homeostatic contexts.

Upon Lipopolysaccharide (LPS) stimulation, SRC-3 suppresses inflammation by repressing translation of pro-inflammatory cytokines. Macrophages lacking SRC-3 show elevated TNFα, IL-6, and IL-1β protein levels without corresponding mRNA changes. Mechanistically, it has been shown that SRC-3 recruits translational repressors to AREs in the 3′ UTR of TNF mRNA (Fig. 4C) (113). Supporting this mechanism, elevated levels of TNFα, IL-6, and IL-1β proteins were observed in SRC-3 KO mice following *E*scherichia *coli*–induced peritonitis, despite unchanged transcript levels of these genes (114). Similar to SRC-3, SRC-2 also contributes to the suppression of pro-inflammatory cytokines during pathological inflammation. Specifically, SRC-2 partners with the GR to suppress the expression of IL-1α, IL-1β, TNFα, and CCL4 in bone marrow–derived macrophages (BMDMs) following LPS stimulation (115) (Fig. 4C). This repression is associated with the coordinated recruitment of GR and SRC-2 to NF-κB response elements bound by the p65 subunit, implying GR-SRC-2-mediated repression of the NF-κB-driven inflammatory program. Transcriptomic profiling further demonstrated that SRC-2 significantly contributes to GR-dependent repression of inflammatory responses, with over 60 of the 152 genes normally suppressed by Dex following LPS exposure being re-expressed in SRC-2-deficient, but not in WT BMDMs. In line with these *in vitro* findings, SRC-2 KO in macrophages increased susceptibility to LPS-induced endotoxicity *in vivo*, underscoring the critical role of SRC-2 in regulating macrophage-mediated inflammatory responses.

Interestingly, under anti-inflammatory conditions induced by IL-4, SRC-2 promotes the adoption of an M2-like anti-inflammatory phenotype in BMDMs via a GR-independent mechanism (116); In WAT, SRC-2 is essential for maintaining the balance between pro-inflammatory monocyte-derived M1 macrophages and tissue-resident anti-inflammatory M2 macrophages, facilitating a shift toward tissue-protective, anti-inflammatory states. Loss of SRC-2 in myeloid cells disrupts this balance, leading to increased M1-driven inflammation, impaired insulin signaling, and reduced glucose tolerance (116). These findings highlight the context-dependent roles of SRC-2 in driving anti-inflammatory programs in macrophages: in pro-inflammatory settings, SRC-2 partners with GR to suppress NF-κB-driven transcription in M1 macrophages, whereas in anti-inflammatory conditions, it supports M2 polarization independently of GR by acting as a coactivator of the TF Krüppel-like factor (KLF)-4—a known integrator of IL-4 signaling in macrophages (Fig. 4C) (117).

#### Additional roles in immunity

All 3 SRC family members physically interact with and coactivate the transcriptional activity of the major inflammatory regulator NF-κB (96). This partnership between SRCs and NF-κB is particularly evident in immune-related contexts. For example, SRC-1 cooperates with the NF-κB subunit p65 to activate the promoter of the pro-inflammatory gene *IL-6* under both basal and angiotensin II–induced inflammatory conditions (118). Similarly, under inflammatory-like conditions induced by TNFα, both SRC-1 and SRC-2 enhance NF-κB transcriptional activity, demonstrating functional redundancy (119). Moreover, as shown in HeLa cells, SRC-3 further amplifies TNFα-induced NF-κB activity in a dose-dependent manner (120). SRCs have a role in the regulation of T and B lymphocyte proliferation; B and T cells isolated from SRC-3 KO mice display increased *ex vivo* proliferation compared with WT counterparts, indicating a cell-autonomous inhibitory role for SRC-3 in lymphocyte expansion. This antiproliferative effect is thought to result from SRC-3's interaction with inhibitor of κB (IκB) kinase, which interferes with IκB phosphorylation and thereby suppresses NF-κB-driven transcription of genes that promote proliferation and survival (121). In murine hematopoietic stem cells (HSCs), SRC-3 deficiency leads to the activation of genes regulated by PGC-1α, which enhances mitochondrial oxidative phosphorylation in HSCs, leading to disruption of their normal function and compromising hematopoiesis. The regulation of mitochondrial metabolism by SRC-3 appears to be essential for preserving the quiescent state of HSCs, and its loss results in increased HSC proliferation. These observations highlight an unanticipated yet crucial role for SRC-3 as a negative regulator of proliferation during early immune cell development (122). In natural killer (NK) cells, SRC-3 is essential for the expression of several key target genes of the master TF T-bet, which govern NK-cell differentiation and effector function—including PRDM1, S1PR5, and Zeb2 (123). Loss of SRC-3 impairs NK-cell maturation and compromises their antitumor function, as evidenced by reduced levels of cytotoxic molecules such as granzyme B and perforin, along with decreased production of interferon-gamma (IFNγ). Mechanistically, ChIP sequencing demonstrated that the recruitment of SRC-3 to promoter regions of key T-bet-binding sites in WT NK cells is dependent on the presence of T-bet (123). SRC-2 plays a critical role in the development and function of CD4⁺ T cells by upregulating solute carrier family 7 member 5 (Slc7a5) (124), an amino acid transporter required for extracellular amino acid uptake, thereby supporting cytokine production, activation, and proliferation upon CD4⁺ T-cell stimulation (125). cKO of SRC-2 in CD4⁺ T cells results in weakened immune responses and protection from autoimmune conditions, as shown in experimental models of colitis and experimental autoimmune encephalomyelitis (EAE) (124).

## SRCs in hormone-dependent cancers and therapy resistance

Although SRCs can function as transcriptional repressors in specific contexts (113, 126), their primary role is to activate gene expression. Deregulation of SRC activity is commonly linked to cancer, and they are traditionally classified as oncogenes (64, 127). While their oncogenic activity is most well established in hormone-dependent cancers—reflecting their primary role as coregulators of hormone-responsive NRs—their oncogenic functions extend beyond hormone-related malignancies. In the following section, we focus on the roles of SRCs in hormone-related cancers, while directing readers to other articles for a discussion of their functions in non-hormone-related cancers (64, 128, 129).

### Breast cancer

#### Steroid receptor coactivator 1

In ERα^+^ breast cancer (BC), SRC-1 acts as an oncogene through regulation of ERα-induced levels of stromal cell–derived factor 1 alpha (SDF-1α; also known as CXCL12), a cytokine that controls BC cell proliferation and invasion (130). In hormone receptor negative BC, SRC-1 expression positively correlates with HER2 expression and poor prognosis (131). In the mouse mammary tumor virus (MMTV)–polyoma middle T antigen (PyMT) BC model, loss of SRC-1 does not impair primary tumor initiation or growth but significantly reduces lung metastases (132). Mechanistically, SRC-1 enhances metastatic potential by promoting cell migration and invasion via activation of Twist expression, achieved through its interaction with the TF polyoma enhancer activator 3 (PEA3) (133), highlighting the role of SRC-1 in driving metastasis also in non-hormone-dependent BC. Beyond its primary role in supporting metastases development, SRC-1 drives therapy resistance in BC by promoting signaling pathways that bypass hormone dependency (134). For instance, SRC-1 promotes resistance to aromatase inhibitors (AIs) by coactivating Ets2-driven transcription of c-myc and MMP-9, further supporting tumor progression independent of hormonal signals (135). SRC proteins are often considered master transcriptional regulators, in part due to their ability to integrate the transcriptional activity of numerous transcription factors, extending well beyond just NRs (136). It was reported that members of the STAT family of TFs, including STAT1, which has been linked to endocrine treatment resistance (137), were coactivated by SRC-1 (138-141). Another pathway by which SRC-1 drives ERα-independent oncogenicity in BC is through regulation of ADAM22, a member of the ADAM protein family, which is well established as a promoter of cancer progression (142-144). In the endocrine-resistant LY2 derivative of the MCF-7 BC cell line, ADAM22 expression was positively correlated with the expression of SRC-1. Moreover, KD of ADAM22 significantly reduced the migratory capacity of both endocrine-resistant and ERα-negative cell lines. These findings highlight the SRC-1–ADAM22 axis as an alternative pathway that drives the shift from steroid-responsive to steroid-resistant BC, enhancing migratory potential and metastatic progression (145). Consistent with this, SRC-1 expression positively correlates with resistance to endocrine therapy and AIs (131, 135, 146, 147). The canonical mechanism by which SRC proteins co-activate NRs involves ligand binding to the NR, which induces conformational changes that enable interaction with coactivators (148). Ligand-independent activation of NRs, on the other hand, can also occur, and it is frequently associated with uncontrolled cell proliferation and cancer (149, 150). It has been demonstrated that cyclin D1, which is frequently amplified in BC, can mediate ligand-independent interaction between ERα and SRC-1 (151). The formation of a transcriptionally active ternary complex (D1–SRC-1–ERα) in the absence of ligand suggests that SRC-1 participates in unregulated activation of ERα in BC.

Aberrant DNA methylation, frequently involving hypermethylation of tumor suppressor genes, is commonly observed in cancer cells (152). Although the majority of SRCs’ activity is associated with transcriptional activation, it has been demonstrated that SRC-1 can also promote oncogenicity by functioning as a global transcriptional repressor (153). Specifically, in endocrine-treatment-resistant models, it has been shown that SRC-1 acts as an integrator in the formation of an epigenetic remodeling complex. SRC-1 mediated recruitment of methyl-CpG-binding domain (MBD) proteins, such as MECP2 and MBD2, which bind methylated DNA and mediate transcriptional repression, along with HDAC2, a known MBD binding partner (154). This coordinated activity leads to the silencing of a set of differentiation-related genes and results in an increased proliferative capacity specifically in endocrine-resistant BC models.

#### Steroid receptor coactivator 2

Steroid receptor coactivator 2 has also been linked to BC tumorigenesis; however, compared with SRC-1 and SRC-3, its role in BC is relatively minor and remains poorly characterized. Like SRC-3, SRC-2 can compensate for SRC-1 deficiency and restore the expression of an ERα target gene, SDF-1α, in the BC cell line (130). Two other studies demonstrated that in MCF-7 cells the expression of pS2, another ERα-responsive gene, as well as DNA synthesis and full-scale ERα transcriptional activity, also require SRC-2 (155, 156). These observations imply the supportive role of SRC-2 in an ERα-driven proliferative signaling in cancer cells. Treatment with tamoxifen in ERα-positive BC patients induces the expression of all 3 SRCs (157), suggesting that like SRC-1 and SRC-3, SRC-2 may also contribute to the development of hormone-therapy resistance. Two recent studies demonstrated a more direct oncogenic role for SRC-2 in BC cells. One study reported that the *NCOA2* gene is amplified in 5% to 14% of BC cases and is essential for BC cell growth, including a triple-negative subtype, suggesting functions beyond ERα signaling (158). Indeed, the authors of this study showed that *NCOA2* perturbation in the TNBC cells MDA-MB-231 led to reduced MAPK/ERK signaling. In a separate study, SRC-2 was implicated in epithelial–mesenchymal transition (EMT) regulation through its control of Lyn kinase, a known EMT marker (159). Depletion of SRC-2 altered the transcriptional program of MCF-7 cells toward a more differentiated luminal gene expression signature, accompanied by reduced expression of genes associated with invasion, suggesting that SRC-2 may contribute to maintaining invasive potential even within luminal BC cells (159).

#### Steroid receptor coactivator 3

Steroid receptor coactivator 3 is also referred to as amplified in BC 1, which underscores its overexpression and strong association with ER-positive BCs (160).

Amplification and overexpression of SRC-3 have been observed in ∼5% to 10% and 30% to 60% of BC biopsies, respectively (46, 161). Both alterations are associated with adverse clinical outcomes, including tamoxifen resistance, aggressive disease, and poor prognosis (162, 163). Mutations in the ligand-binding domain (LBD) of the ESR1 gene generate ERα variants with ligand-independent activity, conferring resistance to AIs and reducing sensitivity to some selective ER degraders (SERDs), such as fulvestrant (164). Although the newer SERDs elacestrant and imlunestrant significantly prolong progression-free survival in ESR1-mutant BC patients, durable long-term remission remains a clinical challenge (165, 166). Unlike the WT ER, whose interactions with SRCs in normal physiological conditions are for the most part estrogen dependent, mutant ER variants can recruit SRC-3 even in the absence of a ligand (12, 167). These ligand-independent interactions drive persistent oncogenic transcription, highlighting the critical role of SRC-3 as an oncoprotein in ER-positive BCs. Interestingly, SRC-3 overexpression in BC has been associated with the absence of estrogen and PRs, suggesting that SRC-3 may also drive tumorigenesis independently of its canonical NR partners, or potentially without engaging NRs at all (168, 169).

Cyclin D1 is a key regulator of the cell cycle, enabling the transition from the G1 to the S phase. In ER-positive BCs, overexpression of Cyclin D1 drives mitogenic signaling and is associated with poor prognosis (170). ER and Cyclin D1 interact in a positive feedback loop, enhancing each other's mitogenic functions (171-174). Evidence from ER-positive BC cells shows that estradiol (E2) induces Cyclin D1 overexpression (173, 175). Importantly, SRC-3 contributes to BC progression by coactivating ER, a function that is essential for ER-mediated induction of Cyclin D1 expression (176).

Several mouse models have further established SRC-3 as an oncogene by demonstrating its critical impact on BC development *in vivo*. In MMTV–v-ras transgenic mice, loss of SRC-3 markedly reduced tumor incidence and completely prevented tumor formation in ovariectomized animals (177). Similarly, in the MMTV–Erbb2 mice model, ERBB2-driven mammary tumor development was entirely suppressed in the absence of SRC-3, highlighting its requirement for ERBB2 oncogenic signaling (178). Furthermore, SRC-3 overexpression, modeled by MMTV–SRC3 transgenic mice, was sufficient to drive spontaneous malignant mammary tumor formation (179). Consistent with SRC-3’s dominant role in the mammary gland, in a chemically induced carcinogenesis model, SRC-3 deficiency protected the mammary tissue, but not the skin, from tumorigenesis (180). Using the MMTV–PyMT animal model SRC-3 has also been shown to promote BC metastasis by mediating the enhanced expression of MMP-2 and MMP-9 through coactivation of the TF PEA3 (181). Interestingly, a study using human BC cells showed that SRC-3 regulates MMP-7 and MMP-10 expression through activation of the TF AP-1 (182), underscoring its role in promoting metastasis by enhancing cancer cell invasion through upregulation of MMPs.

Collectively, this body of evidence establishes all 3 SRCs as key oncogenes in BC tumorigenesis through their roles in tumor initiation, progression, and metastasis.

### Prostate cancer

#### Steroid receptor coactivator 1

Steroid receptor coactivator 1 functions as an important coactivator of androgen receptor (AR). It has been shown that male mice lacking SRC-1 exhibit reduced responsiveness to androgen stimulation and that SRC-1 deficiency results in smaller prostate glands compared with WT controls (65). Additionally, SRC-1 coactivation of AR supports AR-driven cell proliferation involved in prostate development and tissue regeneration (183). In prostate cancer, increased expression of SRC-1 is correlated with aggressiveness of the disease as evidenced by higher levels of SRC-1 in metastatic lesions compared with primary tumors (184). *In vitro* studies have also demonstrated that SRC-1 drives the invasive behavior of prostate cancer cells, independently of AR expression (185). Additionally, in samples from prostate cancer patients, SRC-1 expression levels were higher in tumor tissues compared with normal prostate tissues (185). Interestingly, in a spontaneous *in vivo* prostate cancer mouse model, SRC-1-deficient mice exhibited tumor development similar to that of WT mice. However, in both SRC-1 KO and WT mice, prostate cancer development was accompanied by SRC-3 overexpression, suggesting a potential redundancy in driving the disease in WT animals and a compensatory role for SRC-3 in SRC-1-deficient mice (186). Nonetheless, despite the apparent nonessential role of SRC-1 in tumorigenesis in the spontaneous prostate cancer transgenic model, findings in human samples suggest that SRC-1 plays a critical role in promoting the development and aggressiveness of the disease.

#### Steroid receptor coactivator 2

Steroid receptor coactivator 2 serves as the primary coactivator of AR and is the most strongly linked SRC family member to prostate cancer progression and metastasis (187, 188). Genomic amplification of SRC-2 occurs in ∼8% of primary and 37% of metastatic prostate cancers (189). Beyond its oncogenic role, SRC-2 is a key regulator of energy homeostasis (95, 190) and has been shown to promote metastasis by reprogramming prostate cancer cell metabolism. Activated by glutamine-driven mTORC1 signaling, SRC-2 coactivates the TF sterol regulatory element-binding protein 1 to enhance *de novo* lipogenesis. Consistent with this metabolic role, disruption of SRC-2 function has been found to impair prostate cancer growth and metastases (191).

#### Steroid receptor coactivator 3

Like the other 2 SRC family members, SRC-3 is also strongly involved in AR-related development and progression of prostate cancer (192, 193). Consistent with its strong association with coactivation of AR, elevated SRC-3 levels in prostate cancer patients positively correlated with higher tumor grade and increased risk of recurrence (188, 194). Analysis of 58 RNA samples from prostate cancer patients revealed that overexpression of SRC-3 is positively correlated with invasion and metastasis (195). Supporting these findings, *in vitro* assays have shown that SRC-3 plays an essential role in prostate cancer cell motility and invasiveness through the direct induction of key MMPs, MMP-2, and MMP-13, by partnering with the transcription factors AP-1 and PEA3 (195) . At the molecular level, SRC-3 has been shown to cooperate with the transcription factor AP-1 to upregulate gene expression in the insulin-like growth factor/AKT signaling pathway, thereby promoting cancer cell proliferation and survival, including in prostate cancer cell lines (193, 196).

In the spontaneous transgenic adenocarcinoma of the mouse prostate (TRAMP) model, elevated SRC-3 expression was observed in prostate tumor cells as they advanced to more malignant stages. Loss of SRC-3 suppressed tumor growth and progression and significantly prolonged the survival of TRAMP mice (197). These findings indicate that SRC-3 promotes tumor aggressiveness and plays a critical role in the progression of prostate tumorigenesis toward the metastatic stage.

### Female genital cancers

#### Steroid receptor coactivator 1

In normal endometrial tissue, SRC-1 mRNA levels positively correlate with ERα expression (198). In endometrial carcinoma, SRC-1 expression is reduced compared with normal proliferative-phase endometrial tissue, and this decrease in SRC-1 levels is associated with loss of ER and PR expression (199). Conversely, another study has reported that all 3 SRCs have upregulated mRNA levels in endometrial carcinoma relative to normal endometrium. However, no significant correlation was observed between the overexpression of SRCs and key pathological parameters, such as disease stage or invasiveness (198).

#### Steroid receptor coactivator 2

Steroid receptor coactivator 2 is the least studied among the 3 SRC family members in female genital cancers. However, it was reported that both SRC-2 and SRC-3 are elevated in the endometrium of women with PCOS (74, 76), a condition associated with increased risk of developing estrogen-driven endometrial hyperplasia and cancer (200).

#### Steroid receptor coactivator 3

Steroid receptor coactivator 3 has been associated with the pathogenesis of both endometrial and ovarian cancers. In a mouse model with MMTV-driven SRC-3 overexpression, elevated SRC-3 levels were associated with increased endometrial tumor formation (179). In human endometrial tumors, high SRC-3 expression correlates with advanced disease stage, increased myometrial invasion, and unfavorable clinical outcomes (80, 201). Similarly, in ovarian cancer, SRC-3 is often amplified, and its overexpression was found in >60% of high-grade tumors (46, 202). Experimental evidence further indicated that loss of SRC-3 impairs ovarian cancer cell migration and motility (203).

### Therapy resistance

Among the 3 SRC family members, SRC-1 and SRC-3 are most strongly linked to endocrine therapy resistance. Tamoxifen, a selective ER modulator, is a standard antiestrogen therapy for premenopausal patients with ERα-positive BC. Treatment with tamoxifen is associated with increased expression of both SRC-1 and SRC-3 (147, 157). In the T47D BC cell line, overexpression of SRC-1 has been shown to enhance ERα transcriptional activity in response to E2, effectively converting tamoxifen into a transcriptional activator in these cells (204). Furthermore, in MCF-7 cells that acquired tamoxifen resistance after continuous exposure, SRC-1 levels decreased once the tamoxifen treatment was lifted, suggesting that SRC-1 expression is specifically associated with conferring resistance in ERα-positive BC cells during treatment (205). Immunohistochemical analyses of tissue microarray revealed that the co-association of SRC-1 and SRC-3 with ERα is linked to reduced disease-free survival (DFS) and endocrine therapy resistance in patients (134). SRC-1 may also contribute to therapy resistance by its role in regulating ADAM22, a nonprotease member of the ADAM disintegrin family, that was identified as an independent marker of poor DFS (145). In tamoxifen-resistant cell lines, ADAM22 has been associated with cell migration and differentiation, and its mRNA expression is increased in tamoxifen-resistant tumors in xenograft mouse models (145).

Examination of patient samples from ERα-positive BCs revealed that tumors expressing PAX2 but lacking SRC-3 showed the most favorable DFS outcomes compared with other PAX2/SRC-3 expression profiles. Using human BC cell lines, the authors of this study have demonstrated that tamoxifen–ER complexes repress HER2 transcription, whereas SRC-3 competes with the tamoxifen-recruited transcriptional repressor PAX2, leading to HER2 upregulation and promoting HER2-related tumor aggressiveness (145). These findings suggest a possible mechanism in which SRC-3 outcompetes the tamoxifen-recruited transcriptional repressor PAX2, thereby promoting HER2-driven drug resistance in ERα-positive BC.

Aromatase inhibitors suppress estrogen biosynthesis and are widely used to block ERα signaling in postmenopausal women with hormone-dependent cancer. Examination of tumors from AI-resistant patients and the AI-resistant cell-line model revealed a significant association of SRC-1 expression with disease recurrence and drug resistance. SRC-1 was essential for maintaining the enhanced metastatic potential of AI-resistant cells, while its KD restored cellular differentiation and reduced migratory capacity. Interestingly, ERα KD had no significant impact on the migratory capacity of AI-resistant cells, suggesting that SRC-1 mediates resistance and the aggressive phenotype of the disease through steroid-independent mechanisms. In endocrine-resistant BC cells, SRC-1 interacts with other transcription factors, including Ets2—a member of the ETS family and a downstream effector of HER2 and MAPK pathways (131, 135, 206). This suggests an alternative mechanism by which SRC-1 promotes an aggressive disease phenotype while bypassing canonical ERα signaling.

Estrogen receptor α mutations, which typically occur within the LBD, often lead to ligand-independent oncogenic transcriptional activity following AI treatment (207). It has been demonstrated that all SRC family members show enhanced recruitment to estrogen response elements when bound to mutant forms of ERα LBD (12, 208). Moreover, the constitutive transcriptional activity of these mutants is associated with their enhanced ability to recruit coregulators such as SRC-3 (12, 209), suggesting an additional mechanism through which SRCs may contribute to endocrine therapy resistance.

Beyond their primary roles in tumor initiation, progression, and metastasis in hormone-dependent cancers, SRCs also play a dominant role in the development of resistance to endocrine therapy. This adds an additional layer to their oncogenic potential, highlighting the promise of SRC-targeted therapeutic strategies.

## Evolving therapeutic approaches: small molecules and cell therapy

**Figure 5:**
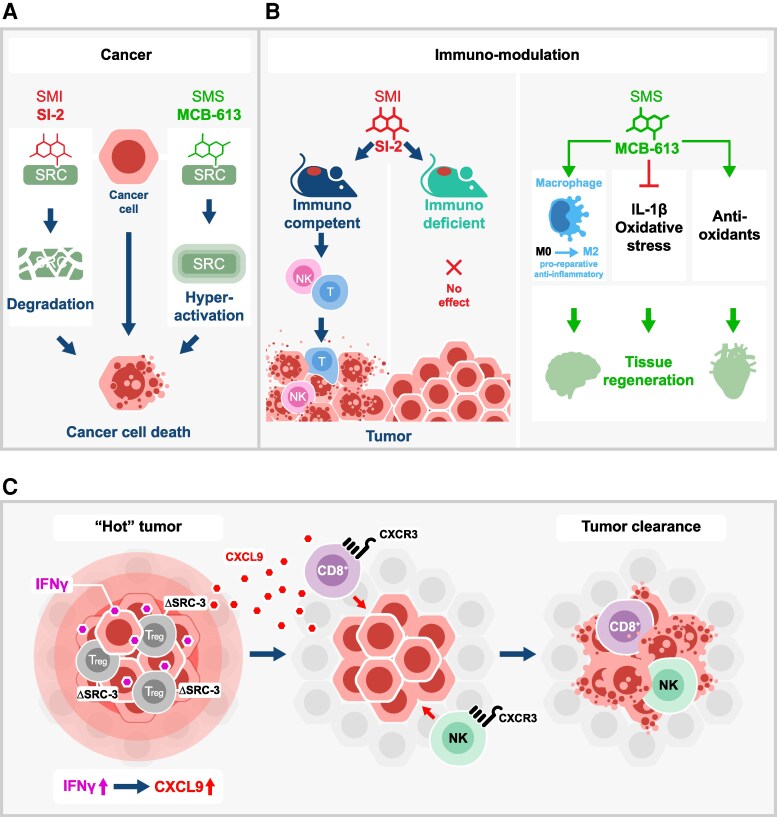
Targeting SRCs: emerging therapeutic strategies: (A) Inhibition or stimulation of SRCs with either SMI or SMS suppresses cancer cell growth: Inhibition of SRCs with SMIs induces their degradation, resulting in reduced proliferation and enhanced apoptosis. In contrast, stimulation of SRCs with SMSs causes hyperactivation of SRC transcriptional activity, triggering cancer cell–specific cytotoxic stress. (B) Immunomodulatory effects of SRC small molecule moderators: SMI SI-2 triggers an antitumor immune response characterized by the secretion of pro-inflammatory cytokines and recruitment of cytotoxic lymphocytes into the TME (left). Stimulation of SRC-3 with SMSs leads to an increased presence of anti-inflammatory macrophages, promoting the establishment and maintenance of a pro-reparative environment that facilitates regeneration of damaged tissue (right). (C) SRC-3 regulates the immunosuppressive function of Tregs: SRC-3 KO in Tregs results in a remodeled, antitumor TME, characterized by increased levels of pro-inflammatory cytokines and enhanced recruitment of cytotoxic lymphocytes, which ultimately drives eradication of solid tumors in mouse models.

As multifaceted oncoproteins, SRCs represent compelling therapeutic targets. Given their pivotal role in modulating hormone receptor activities, targeting SRCs—rather than the NRs themselves—offers a promising alternative, particularly as traditional hormone-based therapies often lose effectiveness due to resistance (127). However, SRCs have long been considered challenging to target pharmacologically due to their structural flexibility and absence of a well-defined LBD. As a result, initial strategies focused on disrupting SRC–NR interactions instead of directly targeting the SRC proteins themselves (23-25). High-throughput screening combined with expression reporter assays led to our discovery of several naturally occurring inhibitors that directly target SRCs (26, 210, 211). This platform ultimately enabled the identification of the first-generation synthetic compounds that directly target the SRCs and bring about either inhibition (SMIs) (27) or stimulation (SMSs) (28) of their activity (Fig. 5A). For example, the SMI SI-2 has been shown to physically interact with SRC-3, leading to a reduction in SRC-3 protein levels in cancer cells. By depleting this oncoprotein, SI-2 selectively induced cytotoxicity in cancer cell lines *in vitro* and suppressed tumor growth *in vivo* (27). The successful development of synthetic small molecules that directly target SRCs paved the way for second-generation SMIs with improved drug-like properties (212, 213).

Further advancements in SRC SMI-based cancer therapy have come from exploring combination strategies with other molecular targets. One study demonstrated that combining SRC SMIs with CDK4/6 inhibitors not only synergistically enhanced cancer cell killing but also reduced the expression of immunosuppressive markers in surviving cells, potentially reducing immune evasion (214). In another study, a genome-wide screen in ER⁺ MCF-7 cells identified a combinational treatment that enhanced sensitivity to the derivative of SI-2 known as SI-12 across multiple types of cancer cell lines, suggesting pan-anticancer potential that can be achieved by SRC inhibition. Notably, the transcriptional signature of SI-12 differed from that of the ERa inhibitor ICI, indicating distinct mechanisms of action and supporting SRC SMIs as alternatives to traditional hormone therapies (215).

Initially, there were concerns that the SRC stimulator molecules might activate dormant cancer cells and promote tumor growth (16). Surprisingly, the SRC SMS MCB-613 showed inhibitory effects on cancer cells both *in vitro* and *in vivo*. This anticancer effect likely results from the fact that cancer cells already overexpress SRCs and exhibit elevated activation of other growth-promoting genes; further stimulation by SMS overwhelms these cells by inducing stress responses and ultimately suppresses their growth (28).

Consistent with anti-inflammatory roles of SRCs in macrophages, it has been demonstrated that a pan-SRC stimulator, MCB-613, promotes a pro-reparative environment in injured cardiac tissue, highlighting the potential therapeutic applications of SRC targeting beyond cancer (Fig. 5B) (216). Specifically, SRC pharmacological activation induces an anti-inflammatory macrophage phenotype, marked by increased infiltration of CCR2⁻ macrophages expressing the anti-inflammatory and cardioprotective protein TSC22D3 (also known as GILZ) (217, 218). This shift in macrophage composition suggests that the beneficial effects of SRC stimulation are mediated, at least in part, through modulation of macrophage polarization toward a reparative, anti-inflammatory state (216). Furthermore, a derivative of the MCB-613 SRC stimulator with improved drug-like characteristics, named MCB-10-1, was tested for its ability to protect the brain in the setting of ischemic injury, aiming to extend the reparative benefits observed in cardiac tissue to the brain (219). Indeed, pharmacological stimulation of SRCs promoted microglial polarization toward a pro-reparative M2-like phenotype, marked by expression of ARG1—an enzyme recognized for its inflammation-resolving role in macrophages (220, 221). M2-polarized microglia supports neuronal recovery and attenuates neurodegenerative processes (222). Therefore, by promoting M2-microglial phenotype, MCB-10-1 facilitates a neuroprotective response following ischemic injury. These findings suggest that pharmacological activation of SRCs promotes anti-inflammatory macrophages, suppresses pro-inflammatory and apoptotic pathways such as IL-1β signaling and oxidative stress, and induces antioxidant transcriptional programs, highlighting the potential value of SRC SMSs in regenerative medicine (216, 219, 223) (Fig. 5B).

Intriguingly, the antitumor activity of the SMI SI-2 observed in immunocompetent mice was lost in immunocompromised models, suggesting that its therapeutic effect involves not only inhibition of SRC-3's oncogenic activity in tumor cells but also modulation of the tumor immunity (224). Indeed, low-dose treatment with SI-2 induced a moderate cytokine response, bringing about increased infiltration of cytotoxic immune cells and decreased presence of Tregs within the TME, establishing a direct link between SRC-3 inhibition and the activation of antitumor immunity, characterized by increased levels of cytokines with known anticancer functions—such as IFNγ and CXCL9 (225, 226). These observations led to the hypothesis that selectively targeting SRC-3 in Tregs could enhance antitumor immune responses. Indeed, using a syngeneic BC mouse model, durable tumor eradication was achieved following cKO of SRC-3 in Tregs induced by tamoxifen-activated Cre recombinase (227). Moreover, a similar outcome was observed in mouse models of prostate, glioblastoma, lung, colon, and pancreatic cancers, suggesting that SRC-3-deficient Tregs may exert a TCR-independent, pan-cancer therapeutic effect (16, 227). SRC-3 KO in Tregs led to increased infiltration of cytotoxic immune cells into the tumor, along with elevated levels of IFNγ and CXCL9 within the TME. Based on these findings, the proposed mechanism of the antitumor immunity promoted by SRC-3 KO Tregs relies on tumor-specific accumulation of the edited Tregs, which secrete IFNγ within the TME. This increased IFNγ then stimulates tumor and stromal cells to produce CXCL9 (228) to bring about the recruitment of CXCR3-expressing cytotoxic immune cells—such as CD8⁺ T cells and NK cells—into the tumor, thereby promoting tumor clearance (Fig. 5C). Remarkably, when cancer cells were reintroduced into mice previously that had achieved long-term remission from BC after SRC-3 KO Tregs treatment, new tumors failed to form, indicating a durable immune memory response. Notably, SRC-3-deficient Tregs isolated from tamoxifen-treated SRC-3f/f:FOXP3Cre-ERT2/+ transgenic mice can be adoptively transferred into tumor-bearing animals, resulting in an antitumor effect similar to genetically engineered mice. This underscores the translational potential of developing SRC-3 KO Treg-based cell therapy. To support clinical translation, a protocol was developed for generating SRC-3-deficient human Tregs using CRISPR-Cas9-mediated gene editing. This was achieved by introducing Cas9-sgRNA ribonucleoprotein complexes into Tregs via a standard nucleofection procedure, enabling efficient and precise KO of SRC-3 (229). Importantly, the anticancer effect of SRC-3 KO Tregs was not accompanied by any detectable adverse side effects. The absence of autoimmune complications can be attributed to the preferential accumulation of the SRC-3 KO Tregs within the TME, where they secrete IFNγ and trigger the IFNγ-CXCL9 signaling cascade (227). However, the mechanisms underlying the biased accumulation of SRC-3-deficient Tregs in the TME remain incompletely understood and warrant further investigation.

Overall, once considered “undruggable” targets, SRCs were made targetable through an extensive discovery approach that led to the development of synthetic molecules capable of either stimulating or inhibiting their activity. Initially designed for anticancer applications, these molecules have also proven applicable for immune modulation, with potential uses in regenerative medicine. Furthermore, immune-stimulatory effects observed in tumor-bearing mice after treatment with the SRC SMI SI-2, ultimately led to the discovery of antitumor immunity conferred by SRC-3-deficient Tregs *in vivo*, suggesting the potential for developing cell-based therapeutic strategies for solid tumors.

## Summary and prospectives

Steroid receptor coactivators mediate the transcriptional regulation of virtually all NRs and many other TFs. They perform diverse functions in human biology and disease, including key roles as oncogenic drivers, metabolic regulators, central controllers of the reproduction system, and modulators of immune responses. Since the establishment of the coregulation concept and discovery of the first SRC family member, the vast scope of SRC-mediated regulatory functions has rapidly been extensively characterized, positioning them as compelling therapeutic targets, especially in oncology. Initially, due to their “undruggable” nature, early drug discovery efforts focused on identifying ligands that could disrupt the interface between SRCs and their main target proteins, namely NRs. These efforts generated large public libraries of compounds that interfered with NR–coactivator interactions, though it was unclear whether the compound targeted the NR or the coactivator. Nevertheless, this laid the groundwork for more focused screening efforts aimed at identifying ligands that directly target SRCs, ultimately leading to the discovery of several naturally occurring SMIs, followed by the development of synthetic molecules with improved pharmacological properties. In addition to inhibitors, SMSs of SRCs have also been identified. While initially raising concerns, due to SRCs’ well-established oncogenic functions, it was later shown that the SRC stimulator MCB-613 exerts paradoxical anticancer effects. By hyperactivating SRCs—which, along with other growth factors, are already overexpressed and operating at maximal capacity in many cancers—MCB-613 overwhelms cancer cells—inducing lethal levels of cellular stress and ultimately triggering cell death.

In addition to their roles as oncogenes and in canonical hormone-governed physiological processes such as reproduction and metabolism, SRCs are increasingly recognized for their significant functions in immunology—a field where their biology is actively investigated but not yet fully characterized. Nonetheless, their involvement in specific immune cells and immune-related processes is well established, particularly in regulating macrophage activity and the proliferation, differentiation, and function of lymphoid cell subsets, including a key role in governing the Treg–Th17 transition axis. The significance of SRCs in Th17-driven autoimmunity is demonstrated by the resistance to EAE observed in SRC-1- and SRC-3-deficient mice (100, 101). SRC-1 and SRC-2 play contrasting roles in directing CD4⁺ T-cell differentiation between the Treg and Th17 lineages. SRC-1 favors a transition toward an inflammatory phenotype by promoting Th17 cell development over Treg formation, while SRC-2 facilitates the commitment of naive CD4⁺ T cells to the regulatory phenotype. In macrophages, SRCs act both directly and indirectly to promote anti-inflammatory phenotypes, primarily by activating the expression of various anti-inflammatory factors.

The identification of drug-like molecules capable of directly targeting SRCs marks a major advance in the therapeutic potential of SRC modulation. However, due to the high structural similarity among SRC family members, achieving greater specificity remains a key challenge. The nuanced and selective roles of SRCs in regulating immune system functions underscore the need for the development of highly specific modulators. Such modulators may enable the extension of SRC targeting beyond cancer cell inhibition to broader immunomodulatory applications. Nonetheless, the existing arsenal of SRC small-molecule modulators has provided a foundation for progress in this direction. For example, the observation that an SRC inhibitor, SI-2, can both suppress tumor growth directly and enhance antitumor immune response (224) provided the impetus for developing a potential cell therapy using SRC-3 KO Tregs (227). This approach has shown highly promising preclinical results in achieving long-term remission in several solid tumors (227). Moreover, previous observations indicating that SRCs contribute to reparative processes (230) and regulate anti-inflammatory programs in key regenerative cells—particularly macrophages—have supported the idea of extending SRC-targeted therapies beyond oncology. The therapeutic potential of the SMSs MCB-613 and MCB-10-1 in regenerative medicine has been demonstrated in preclinical models of tissue repair following MI and ischemic brain injury. These pro-reparative effects are likely driven by the SMS-related stimulation of SRCs in macrophages, where SRCs are predominantly associated with promoting an anti-inflammatory phenotype.

Overall, current data highlight the strong and diverse therapeutic potential of targeting SRCs. Continued efforts to develop more selective SRC inhibitors will likely build on early successes and broaden their applications. For example, given the well-established roles of SRCs in metabolic regulation and the first clinical trial investigating SRC-1 deficiency as an indicator of metabolic disorders, SRC modulation may offer novel therapeutic strategies for metabolic diseases such as diabetes. Moreover, based on encouraging data showing SMS-mediated pro-reparative shaping of microglia and the central role of these cells in neurodegeneration, targeting SRCs in microglia holds promise for treating neurodegenerative diseases like Alzheimer's disease. Further exploration of SRC modulation in additional immune subsets where they exert dominant biological functions, such as Th17 cells, may open new therapeutic opportunities, including the treatment of autoimmune disorders. Promising preclinical results demonstrating the therapeutic potential of SRC-3 KO Tregs in solid tumors highlight an opportunity to leverage advances in cell-based and gene-editing technologies for the clinical translation of SRC-3-targeted cellular therapies. Together, these insights underscore the vast and still-unfolding therapeutic potential of SRC modulation, fueling strong optimism for future breakthroughs in oncology, immune-related disorders, and beyond.

In summary, the early reasoning that NRs require assistant proteins to achieve the full scale of their biological activity led to the establishment of NR coactivation as a core branch of molecular biology, ultimately culminating in the discovery of one of the most important gene regulatory proteins—the SRCs. Decades of fundamental research have now been translated into challenging yet exciting opportunities for the development of new therapeutic approaches for unmet medical needs.

## Abbreviations

AI: aromatase inhibitor
AR: androgen receptor
BAT: brown adipose tissue
BC: breast cancer
BMDM: bone marrow–derived macrophage
CARM1: coactivator-associated arginine methyltransferase 1
ChIP: chromatin immunoprecipitation
cKO: conditional KO
DFS: disease-free survival
DKO: double KO
EAE: experimental autoimmune encephalomyelitis
EMT: epithelial–mesenchymal transition
ERα: estrogen receptor α
GR: glucocorticoid receptor
GTF: general transcription factor
HFD: high-fat diet
HMTs: histone methyltransferases
HSC: hematopoietic stem cell
IκB: inhibitor of κB
IFNγ: interferon-gamma
KD: knockdown
KLF: Krüppel-like factor
KO: knockout
LBD: ligand-binding domain
MC4R: melanocortin-4 receptor
MMP-9: matrix metalloprotease 9
MMTV: mouse mammary tumor virus
NCOA: nuclear coactivator
NK: natural killer
NR: nuclear receptor
PCOS: polycystic ovary syndrome
PEA3: polyoma enhancer activator 3
PGC-1α: proliferator-activated receptor gamma coactivator-1 alpha
POMC: proopiomelanocortin
PPARγ: peroxisome proliferator-activated receptor gamma
PTMs: posttranslational modifications
PR: progesterone receptor
PyMT: polyoma middle T antigen
SDF-1α: stromal cell–derived factor 1 alpha
SERDs: selective ER degraders
SMI: small-molecule inhibitor
SMS: small-molecule stimulator
SRC: steroid receptor coactivator
TNFα: tumor necrosis factor alpha
TF: transcription factor
TRAMP: transgenic adenocarcinoma of the mouse prostate
WAT: white adipose tissue
